# Pan-cancer analysis reveals IL32 is a potential prognostic and immunotherapeutic biomarker in cancer

**DOI:** 10.1038/s41598-024-58550-5

**Published:** 2024-04-07

**Authors:** Feng Han, Jianxin Ma

**Affiliations:** Department of Oncology, Lianyungang Oriental Hospital, 57 Zhonghua West Road, Lianyungang, 222042 Jiangsu Province China

**Keywords:** Cancer, Genetics, Immunology, Oncology

## Abstract

Interleukin 32 (IL32) is a pro-inflammatory cytokine that plays a key role in promoting sterile inflammation by modulating immune responses. However, the role of IL32 in various cancers remains unclear. This research aimed to investigate the correlation between IL32 expression and immunity and visualize its prognostic landscape in pan-cancer. We investigated gene expression, genomic alterations, and survival analysis of IL32 in pan-cancer in numerous databases including TCGA, GTEx, cBioPortal, and GDC databases. Tumor immune cell infiltration was assessed using the CIBERSORT computational method as well as the ESTIMATE method to analyze the correlation of IL32 expression with stromal and immune components. Protein–protein interaction analysis was performed in the STRING and GeneMANIA databases, and gene function enrichment was performed by GO set enrichment analysis. Tumor tissues had higher IL32 expression levels than normal tissues. Elevated IL32 expression was associated with poor OS and prognosis. In addition, tumor stemness, TMB, MSI, and immune checkpoint genes were also associated with IL32 expression. Correlations were observed between IL32 expression and B cell, CD4T cell, CD8T cell, neutrophil, macrophage, and DC infiltration in multiple cancers. GO enrichment analysis showed that IL32 expression was associated with cancer pathways, cytokine-receptor interactions, and NOD-like receptor signaling pathways. These findings suggest that IL32 may serve as a biomarker of cancer immune infiltration and poor prognosis, providing new therapeutic targets for cancer treatment.

## Introduction

The development of malignant tumors is related to a variety of factors, and monitoring tumor occurrence, early prevention, and timely control of tumors is important for human health^1,2^. Inflammation plays a crucial role in carcinogenesis and affects the development of malignant tumors and invasive metastasis. Interleukin-32 (IL-32), a newly identified pro-inflammatory cytokine, can be involved in the development of inflammatory diseases and a variety of malignancies^1^. IL-32 was initially identified in activated natural killer (NK) and T cells, and its expression was strongly enhanced by microbes, mitogens, and other pro-inflammatory cytokines^2^. Increasing evidence suggests that IL-32 acts as an amplifier of inflammation through its stimulatory effects on pro-inflammatory cytokines, including IL-1β, IL-6, and tumor necrosis factor-alpha (TNF-a). IL-32 is commonly used as a pro-inflammatory cytokine and is highly expressed in inflammatory disorders and a variety of malignant tumors, where it induces the release of cytokines, which are important for the formation of the inflammatory microenvironment and promote the growth, migration, and invasion of tumor cells^3,4^. The inflammatory microenvironment plays a crucial role in tumorigenesis, and targeting the inflammatory microenvironment has preventive and therapeutic implications for a variety of tumors.

In recent years, the analysis of tumorigenesis and progression in pan-cancer has been increasingly studied. Pan-carcinoma analysis allows the analysis of genes in multiple cancers, in which differences and similarities in the expression of extracted genes are compared^5^, revealing similarities and differences in cancers and mining valuable factors for diagnosis, prognosis, and immunotherapy^6^. Therefore, it is meaningful to further explore the pan-carcinogenic gene profiles. To date, the role of IL32 in pan-cancer remains uncertain. In the present study, based on data mining analysis of various databases, we comprehensively analyzed the expression of IL32 and its association with tumor-infiltrating immune cells and related immune markers and further evaluated the prognostic value of IL32 in predicting survival outcomes. Meanwhile, we systematically observed the relationship between IL32 expression and prognosis, tumor-infiltrating immune cells, relevant immune markers, tumor stemness, microsatellite instability (MSI), and tumor mutational load (TMB), and also applied Gene Ontology (GO) enrichment analysis to explore the biological function of IL32 in pan-cancer. In conclusion, our systematic study of the role of IL32 in human pan-cancer will elucidate the clinical role and potential molecular mechanisms of IL32 in pan-cancer.

## Methods

### Data source and processing

Cancer tissue transcriptional data from the TCGA-Pancancer cohort and normal human tissue data from the Genotype-tissue expression (GTEx) database were downloaded from UCSC Xena (https://xenabrowser.net/)^7^. In this study, the abbreviations of each cancer from the TCGA database were shown in the Table 1. The expression profiles were transferred to transcripts per kilobase million (TPM) format, and the log2(TPM + 1) format data were used for subsequent analysis. Related prognostic information, including overall survival (OS) time, progression-free survival (PFS) time, disease-free survival (DFS) time, and disease-specific survival (DSS) time, were also downloaded from the UCSC Xena database.

**Table1:** Abbreviations of cancers in the TCGA database.

Cohort	Full name
TCGA-ACC	Adrenocortical carcinoma
TCGA-BLCA	Bladder Urothelial Carcinoma
TCGA-BRCA	Breast invasive carcinoma
TCGA-CESC	Cervical squamous cell carcinoma and endocervical adenocarcinoma
TCGA-CHOL	Cholangiocarcinoma
TCGA-COAD	Colon adenocarcinoma
TCGA-DLBC	Lymphoid Neoplasm Diffuse Large B-cell Lymphoma
TCGA-ESCA	Esophageal carcinoma
TCGA-GBM	Glioblastoma multiforme
TCGA-HNSC	Head and Neck squamous cell carcinoma
TCGA-KICH	Kidney Chromophobe
TCGA-KIRC	Kidney renal clear cell carcinoma
TCGA-KIRP	Kidney renal papillary cell carcinoma
TCGA-LAML	Acute Myeloid Leukemia
TCGA-LGG	Brain Lower Grade Glioma
TCGA-LIHC	Liver hepatocellular carcinoma
TCGA-LUAD	Lung adenocarcinoma
TCGA-LUSC	Lung squamous cell carcinoma
TCGA-MESO	Mesothelioma
TCGA-OV	Ovarian serous cystadenocarcinoma
TCGA-PAAD	Pancreatic adenocarcinoma
TCGA-PCPG	Pheochromocytoma and Paraganglioma
TCGA-PRAD	Prostate adenocarcinoma
TCGA-READ	Rectum adenocarcinoma
TCGA-SARC	Sarcoma
TCGA-STAD	Stomach adenocarcinoma
TCGA-SKCM	Skin Cutaneous Melanoma
TCGA-TGCT	Testicular Germ Cell Tumors
TCGA-THCA	Thyroid carcinoma
TCGA-THYM	Thymoma
TCGA-UCEC	Uterine Corpus Endometrial Carcinoma
TCGA-UCS	Uterine Carcinosarcoma
TCGA-UVM	Uveal Melanoma

### Analysis of gene expression

The Gene Expression Profiling Interactive Analysis, version 2(GEPIA2.0) database (http://gepia2.cancer-pku.cn/#index) was applied to conduct the analysis based on TCGA and GTEx samples^8^. The screening criteria in GEPIA2.0 were as follows: *p* < 0.05 and the cutoff of |Log2FC| was 0.1.

### Gene alteration analysis

The cBioPortal database (https://www.cbioportal.org/) was utilized for down load ing mutation and copy number variation (CNV) data of IL32^9^

### Survival prognosis analysis

For exploring the IL32 expression effect on pan-cancer prognosis, we used the R packages “survminer” and “survival” to conduct Kaplan–Meier regression analyses and univariate Cox regression (UniCox) analyses, respectively. The optimal cutoff value was utilized to differentiate the groups of IL32 with low and high expression. Survival analyses (overall, disease-specific, progression-free, and disease-free) were assessed.Cox proportional hazards regression model was used to analyze the prognostic value of IL32 on cancers, and log rank test was used to obtain prognostic significance.

### The clinical correlations of IL32 in the pan-cancer

We calculated the differences in the expression of IL32 in each tumor in samples with different clinical stages using R software. Unpaired Wilcoxon rank sum and signed rank tests were used to perform differential significance analysis and performed tests of differences in multiple samples using the kruskal test. The clinical correlations of IL32 in pan-cancer were evaluated.

### The correlation between IL32 and immune cell infiltration was calculated

Sangerbox 3.0 (http://www.sangerbox.com) is a web-based bioinformatics analysis platform.We used Sangerbox 3.0, utilizing data from the XCELL database to analyze the association between IL32 expression and tumor immune infiltration.

### The correlations of RNA modification, checkpoints and immunoregulatory genes with the mRNA expression of IL32

Using Sangerbox 3.0, we examined the correlations between IL32 and 44 marker genes of three different kinds of RNA modification (10 of m1A, 13 of m5C, and 21 of m6A). Additionally, correlations between the mRNA expression of IL32 and 150 immunoregulatory genes, including chemokine (41), receptor (18), MHC (21), immunoinhibitory (24), and immunostimulatory (46) were conducted.

### Tumor stemness indexes analysis

Using Sangerbox 3.0, we analyzed the relationship between tumor stemness characteristics and IL32 expression, utilizing four types of stemness indices for testing, including those based on differential methylation probes (DMPss), DNA methylation (DNAss), enhancer elements/DNA methylation (ENHss), and epigenetically regulated DNA methylation (EREG-METHss)^10^. We also combined the stemness indices with gene expression data, further transforming each expression value into log2 (x + 1), calculated the Spearman correlation for each tumor sample, and plotted “lollipop charts.”

### TMB and MSI analysis

In different tumors, tumor mutation burden (TMB) is associated with immunotherapy efficacy. TMB obtained from the GDC (https://portal.gdc.cancer.gov/) and proceeded by MuTect2 software and R package “maftools”^11^, MuTect2 from GATK (version4.5.0.0, https://github.com/broadinstitute/gatk/releases) software.Microsatellite instability (MSI)^12^ were used to assess the relationship between tumor heterogeneity and IL32 expression.

### Protein–Protein interaction network construction

STRING (https://string-db.org/) is a database that collects, integrates, and scores nearly all publicly available sources of protein–protein interaction (PPI) data with the goal of annotating functional protein association networks^13^. Using the STRING website, we investigated the proteins that were experimentally confirmed to interact with IL32 and eventually compiled a list of these proteins.

GeneMANIA (http://www.genemania.org), a user-friendly and interactive website for building protein–protein interaction (PPI) networks, develops hypotheses regarding the prediction of gene functions and identifies genes with related activities^14^. In this study, GeneMANIA was applied for PPI analysis of IL32.

### Gene Ontology (GO) enrichment analysis of IL32 in pan-cancer

Then we merged the two gene lists to execute GO enrichment analysis using the R package “clusterProfiler”^15^. The minimal gene set was set to 5, while the largest gene set was 5000. The *p*-value was set to 0.01 and FDR of < 0.25.

### Statistical analysis

We conducted all analyses through GEPIA2.0, cBioPortal, Sangerbox 3.0, STRING, GeneMANIA, R software (version 4.3.3, https://mirrors.tuna. tsinghua.edu.cn/CRAN/) and its suitable packages. Unpaired Wilcoxon rank sum and signed rank tests were used to analyze pairwise differences, and Kruskal test was used to test multiple sets of samples. Statistical significance was set as two-sided *p* < 0.05. Significance was marked as follows: *, *p* < 0.05; **, *p* < 0.01; ***, *p* < 0.001.

### Ethical approval and consent to participate

The authors are accountable for all aspects of the work in ensuring that questions related to the accuracy or integrity of any part of the work are appropriately investigated and resolved.

## Results

### The mRNA Expression and Genetic Alteration Differences of IL32 in Cancers

**Figure 1:**
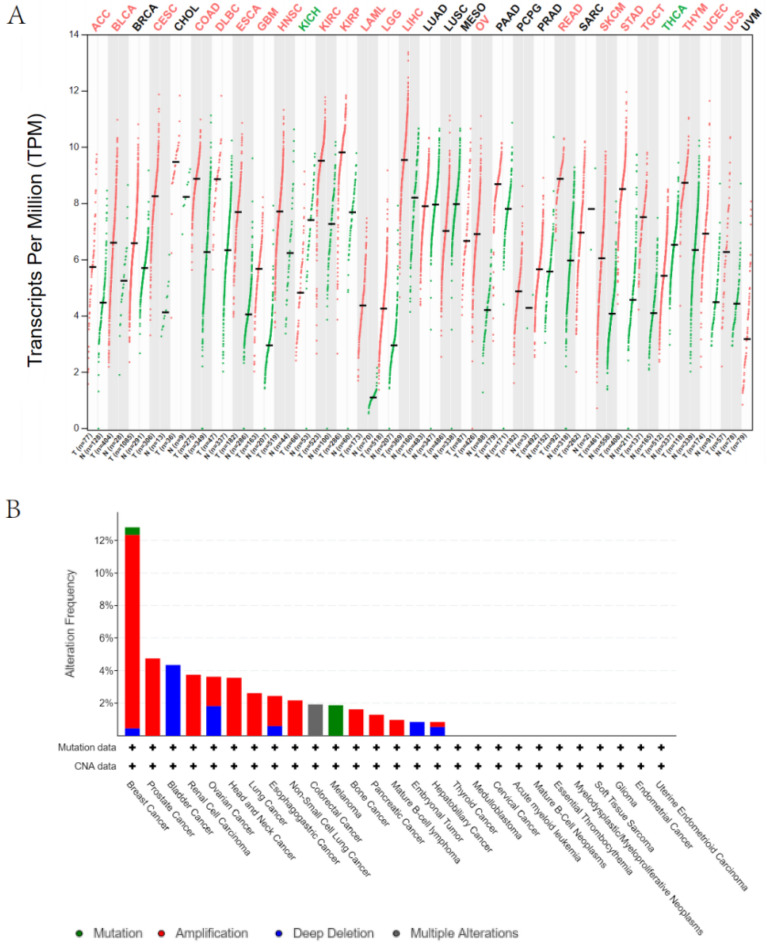
The mRNA Expression and Genetic Alteration Differences of IL32 in Cancers. (**A**) Expression level of IL32 in different cancer types from TCGA and GTEx data. The red fusiformis represents tumor tissue, and the blue fusiformis represents normal tissue. T, tumor; N, normal; n, number. X axis, number of tumor and normal samples. Y axis, transcript per million [log2(TPM + 1)]. (**B**) The genetic alteration type and frequency of IL32 in various cancers. The cBioPortal database was applied to study the IL32 mutation in cancers. The results are displayed as a histogram of the alteration frequencies of IL32 across cancer studies. Color images are available online.

IL32 expression was assessed using TCGA and GTEx data for tumors without normal control. IL32 overexpression was detected in 25 of 33 types of cancer, comprising ACC, BLCA, CESC, COAD, DLBC, ESCA, GBM, HNSC, KIRC, KIRP, LAML, LGG, LIHC, OV, READ, SKCM, STAD, TGCT, THYM, UCEC and UCS. Even so, IL32 under-expression was detected in two tumors, comprising THCA and KICH (Fig. 1A). It has been widely acknowledged that genomic mutation is closely associated with tumorigenesis. To figure out genomic mutation of IL32 in cancers, comparative analysis of IL32 was performed. We firstly checked the genetic alterations of IL32 genes in cancer patients using cBioPortal database. Gene alteration mapping of IL32 showed that IL32 amplification was the most important single altering factor in PRAD, Renal cell carcinoma, HNSC, LUAD, LUSC, Bone cancer, PAAD and DLBC.We observed the highest frequency of IL32 alterations (> 12%) in patients with BRCA, where “Amplification” was the major type (Fig. 1B).

### The pan-cancer analysis of clinical correlation withIL32 expression

**Figure 2:**
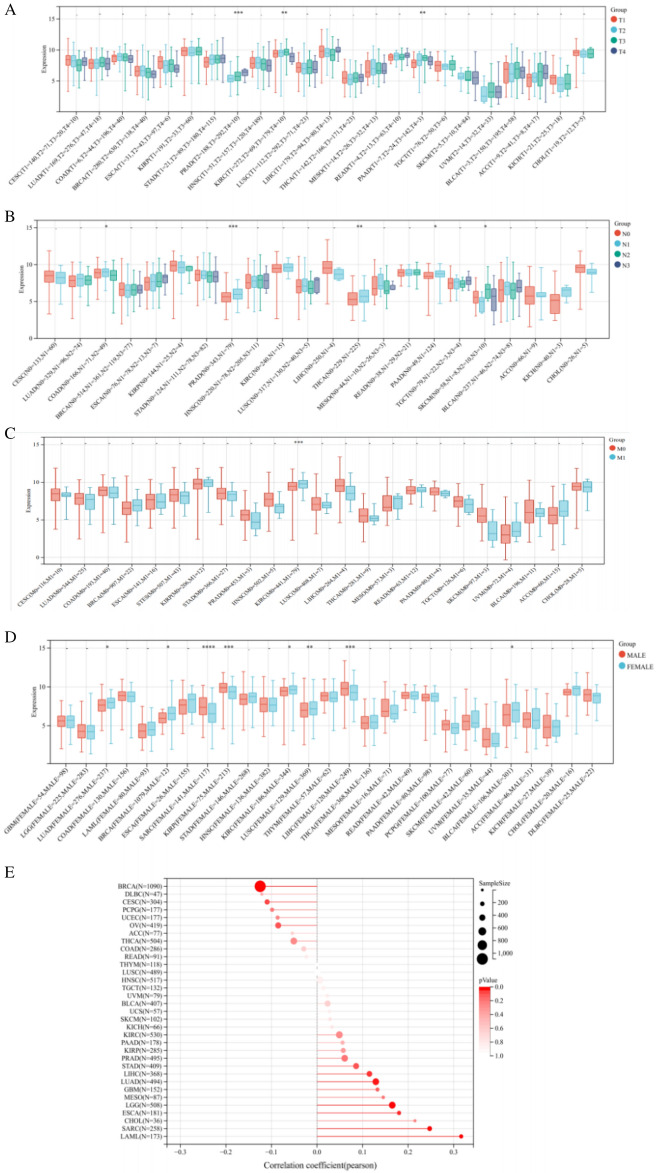
The pan-cancer analysis of clinical correlation with IL32 expression. The correlation ofIL32 expression with (**A**) T stages, (**B**) N stages, (**C**) M stage, (**D**) gender; *P < 0.05, **P < 0.01, ***P < 0.001. ****P < 0.0001. (**E**) The correlation of IL32 expression with age, deeper color indicates a strong correlation.

The correlation of IL32 expression with pathological tumor staging in the TCGA cohort was done, and it was raised as T stages increased in PRAD (*p* = 1.4e-4), KIRC (*p* = 2.6e-3), PAAD (*p* = 1.2e-3) (Fig. 2A). Differential expression of IL32 was significant among N stages for PRAD (*p* = 9.1e-4), THCA (*p* = 8.1e-3), PAAD (*p* = 0.03) and SKCM (*p* = 0.03), as well as for M stages of KIRC (*p* = 1.5e-4) (Fig. 2B). In KIRC (*p* = 9.5e-4), THCA (*p* = 0.01) and PAAD (*p* = 1.4e-6), IL32 expression was elevated with increasing clinical stages (Fig. 2C).Differential expression of IL32 was significant among Gender for LUAD (*p* = 0.03), BRCA (*p* = 0.03), SARC (*p* = 7.0e-5), KIRP (*p* = 8.3e-4), KIRC (*p* = 0.02), LUSC (*p* = 9.7e-3), LIHC (*p* = 2.6e-4) and BLCA (*p* = 0.01) (Fig. 2D). Advanced age was related to elevated IL32 expression in LGG (*P* < 0.0001), LUAD (*P* = 0.004), LAML (*P* < 0.0001), ESCA (*P* = 0.015), SARC (*P* < 0.0001), LIHC (*P* = 0.027) (Fig. 2E).

### IL32 high expression in pan-cancer is related to poor prognosis

**Figure 3:**
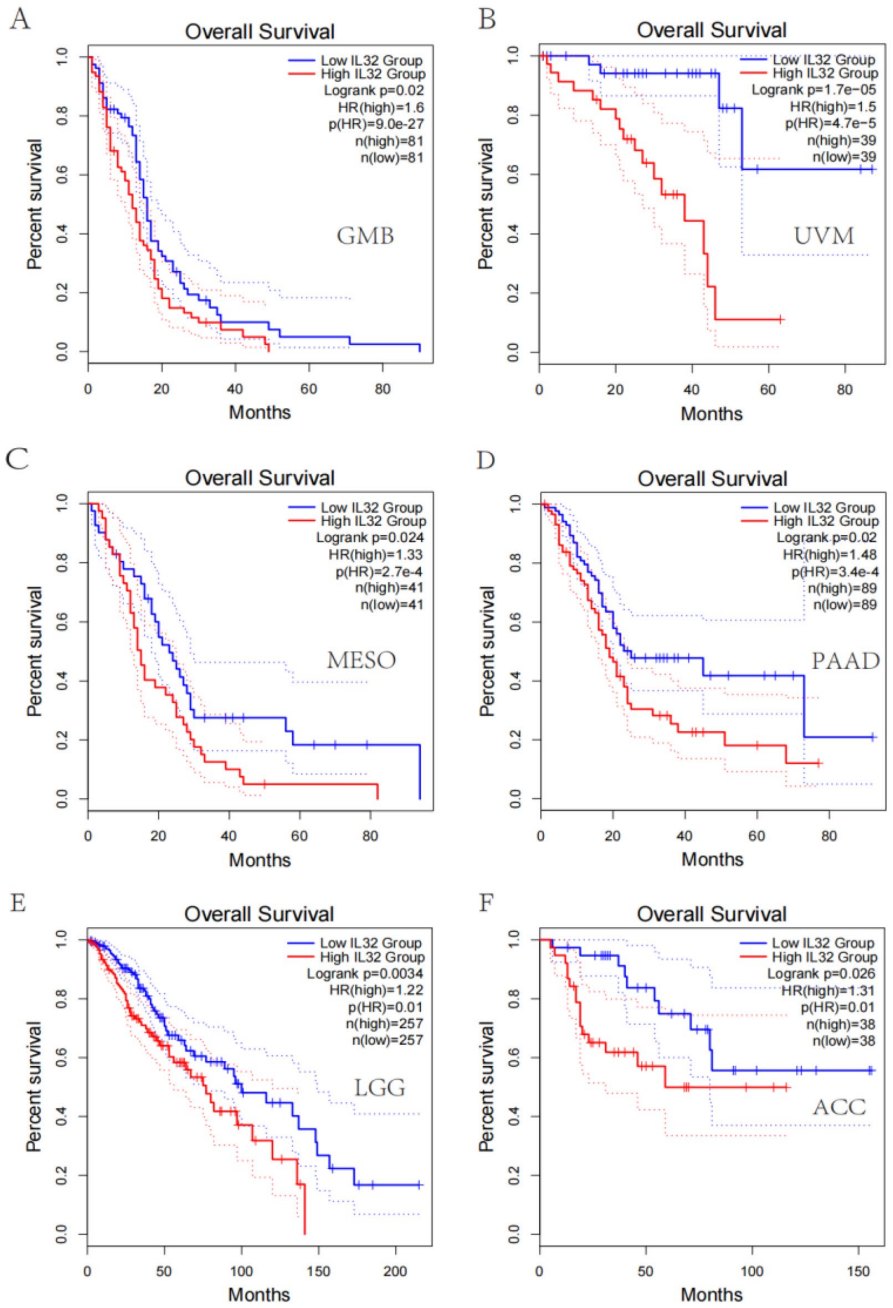
IL32 prognostic value. IL32 overall survival analysis via Kaplan–Meier in LGG (**A**), GBM (**B**), MESO (**C**), UVM (**D**), LAML (**E**), ACC (**F**).

**Figure 4:**
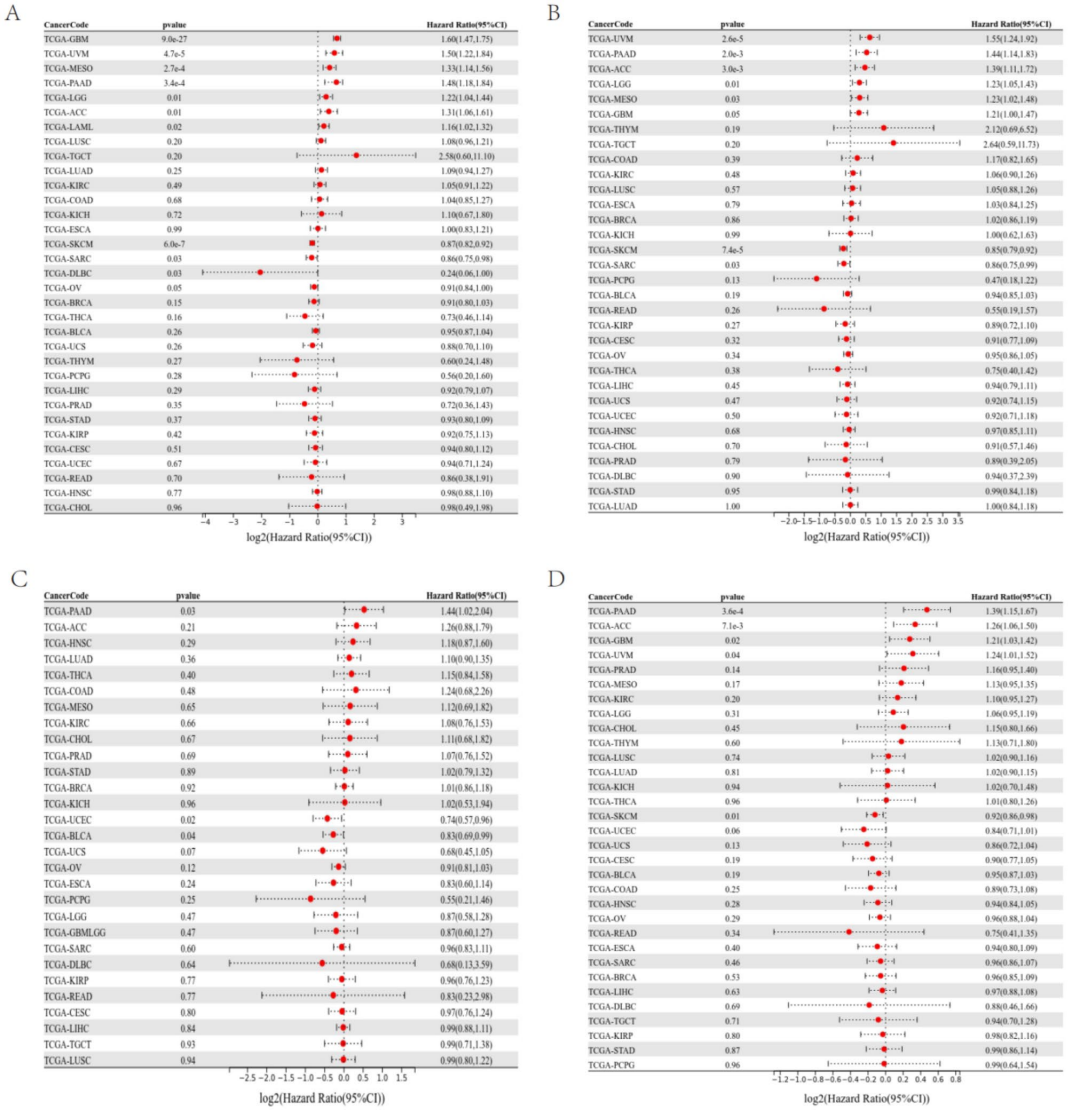
IL32 UniCox analysis. (**A**) IL32 overall survival (OS) analysis in TCGA pan-cancer utilizing the UniCox. (**B**) IL32 disease-specific survival (DSS) analysis in TCGA pan-cancer utilizing the UniCox. (**C**) IL32 disease-free interval (DFI) analysis in TCGA pan-cancer utilizing UniCox. (**D**) IL32 progression-free interval (PFI) analysis in TCGA pan-cancer utilizing UniCox.

The UniCox and Kaplan–Meier survival analyses were utilized for exploring IL32 prognostic value in pan-cancer. IL32 low and high expressions were differentiated using the optimal cut-off value. According to Kaplan–Meier, worse overall survival was related to elevated IL32 expression in LGG (HR = 1.22, *p* = 7.8e-3), GBM (HR = 1.23, *p* = 0.02), MESO (HR = 1.31, *p* = 5.1e-4), UVM (HR = 1.58, *p* = 4.2e-6), LAML (HR = 1.14, *p* = 0.03), ACC (HR = 1.38, *p* = 2.6e-3) (Fig. 3A–F). IL32 was considered to be a risk factor for OS, according to UniCox analysis in UVM, PAAD, ACC, LGG, MESO, GBM, SKCM and SARC (Fig. 4A). IL32 prognostic value in pan-cancer for DSS, DFI, and PFI was also analyzed, and the results are illustrated in Fig. 4B–D. Based on these findings, elevated IL32 expression in pan-cancer was linked to a poor prognosis and might be a potential prognostic biomarker.

### Correlation between IL32 expression and immune infiltrating level in cancers

**Figure 5:**
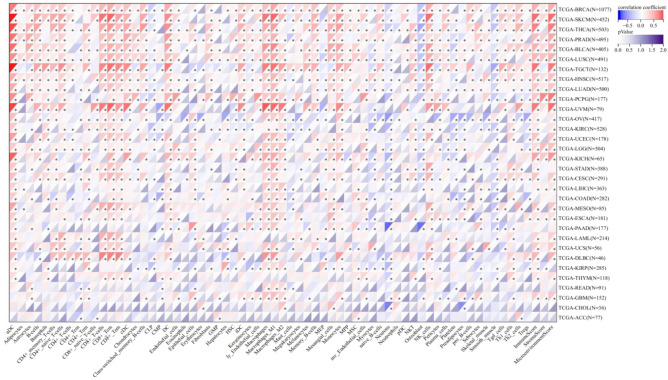
Immune infiltration analysis. Correlation of IL32 expression with immune cell infiltration by XCELL database. Red and blue colors indicate positive and negative correlations, respectively; deeper color indicates a strong correlation *P < 0.05.

The level of tumor immune infiltration is crucial for the survival and immunotherapy of cancer patients. Therefore, we studied the correlation between IL32 expression and the level of immune infiltration in 33 types of cancer using the XCELL database. As shown in Fig. 5, IL32 expression is positively correlated with the immune scores of BRCA, SKCM, THCA, PRAD, BLCA, LUSC, TGCT, HNSC, LUAD, PCPG, UVM, KICH, CESC, DLBC, and is positively correlated with the stromal scores of BRCA, SKCM, PRAD, BLCA, LUSC, HNSC, LUAD, PCPG, LGG, KICH, LIHC, ESCA, GBM, and negatively correlated with the stromal score of KIRP. Additionally, the correlation between IL32 expression and 38 types of immune cell subtypes was analyzed, showing that IL32 expression is significantly positively correlated with most immune cell subtypes in BRCA, SKCM, THCA, PRAD, BLCA, LUSC, TGCT, HNSC, LUAD, PCPG, UVM, KICH, CESC, DLBC, and significantly positively correlated with NK cells, CD4 + memory T-cells, CD8 + T cells, M1, B cells, and dendritic cells.

### IL32 expression is associated with immune checkpoint genes and immune regulatory genes

**Figure 6:**
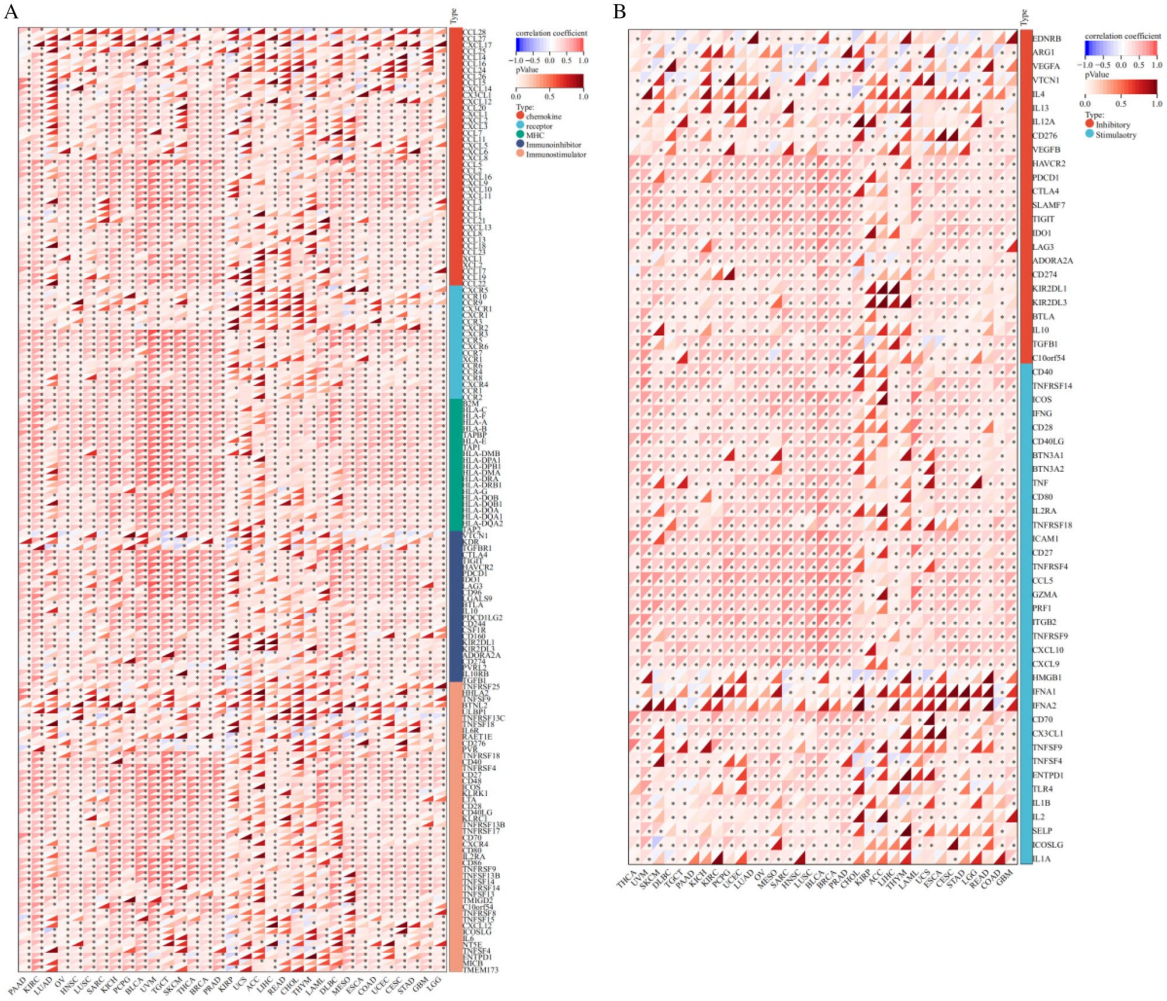
Correlation of IL32 expression with immunomodulators and immune checkpoints. (**A**) Correlation between IL32 and 150 immunomodulators (chemokine, MHC, Immunoinhibitor, Immunostimulator). (**B**) Correlation between IL32 and 60 immune checkpoints. Red and blue colors indicate positive and negative correlations, respectively; deeper color indicates a strong correlation *P < 0.05.

We explored the correlation between IL32 expression and 150 immune regulatory factors (chemokines, MHC, immune inhibitors, immune stimulators) in cancer. Figure 6A shows that, in most cancers, IL32 expression is positively correlated with most immune regulatory factors. At the same time, in most malignancies, there is a positive correlation between IL32 and most immune checkpoint inhibitors, especially in PAAD, DLBC, LUAD, UVM, and THCA (Fig. 6B).

### IL32 correlation with TMB and MSI in pan-cancer

**Figure 7:**
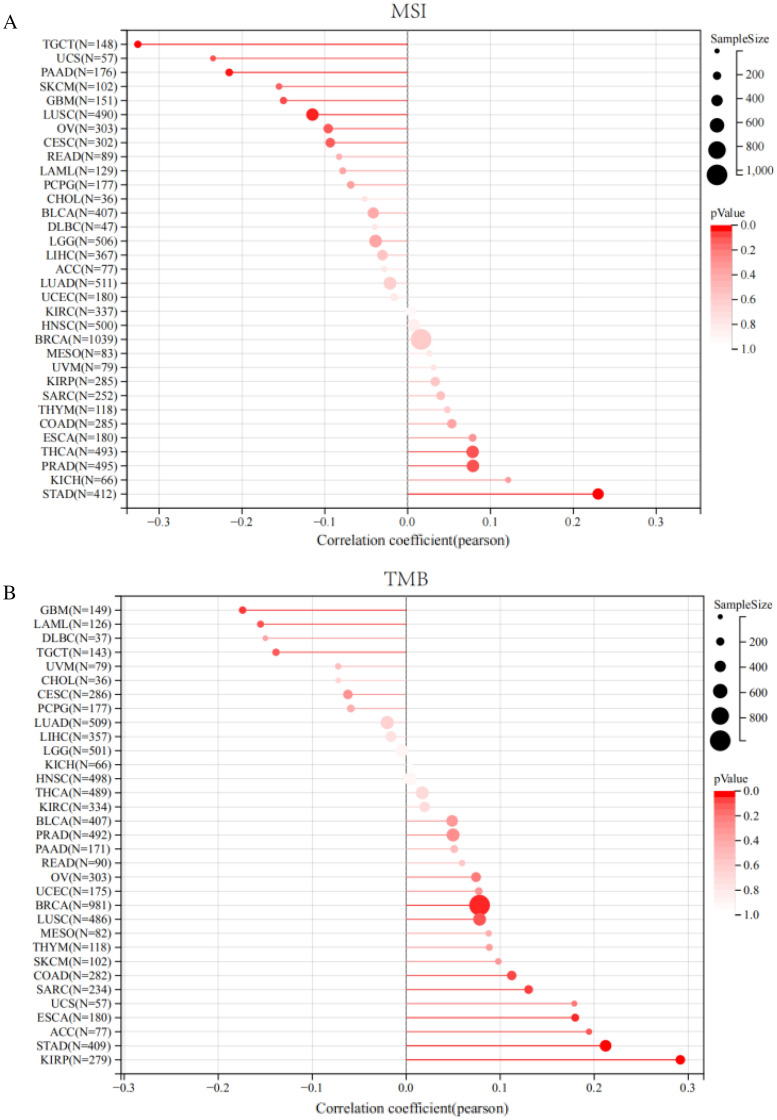
IL32 correlation with tumor mutation burden (TMB) and microsatellite instability (MSI). IL32 correlation with TMB (**A**) and MSI (**B**) of Radar plots in pan-cancer. Red dots indicate the correlation coefficient. Deeper color indicates a strong correlation.

Each tumor sample’s TMB was calculated, and correlation was assessed between IL32 expression and TMB. The results are illustrated in Fig. 7A. The expression levels of IL32 showed a significant positive correlation with TMB in BRCA (R = 0.078, *P* = 0.015), ESCA (R = 0.180, *P* = 0.016), SARC (R = 0.130, *P* = 0.046), KIRP (R = 0.291, *P* = 7.14e-7) and STAD (R = 0.212, *P* < 0.0001), and a negative correlation with TMB in GBM (R = − 0.174, *P* = 0.034). The correlation of IL32 expression with MSI was assessed, and the results are illustrated in Fig. 7B. Notably, IL32 expression levels had a significant positive correlation with MSI in THCA (R = 0.078, *p* < 0.021), PRAD (R = 0.076, *p* < 0.035) and STAD (R = 0.230, *p* < 0.0001), and a significant negative correlation with MSI in LUSC (R = − 0.115, *P* = 0.011), PAAD (R = − 0.215, *P* = 0.004) and TGCT (R = − 0.326, *p* < 0.0001).

### Results from the perspective of tumor stemness and epigenetic regulation

**Figure 8:**
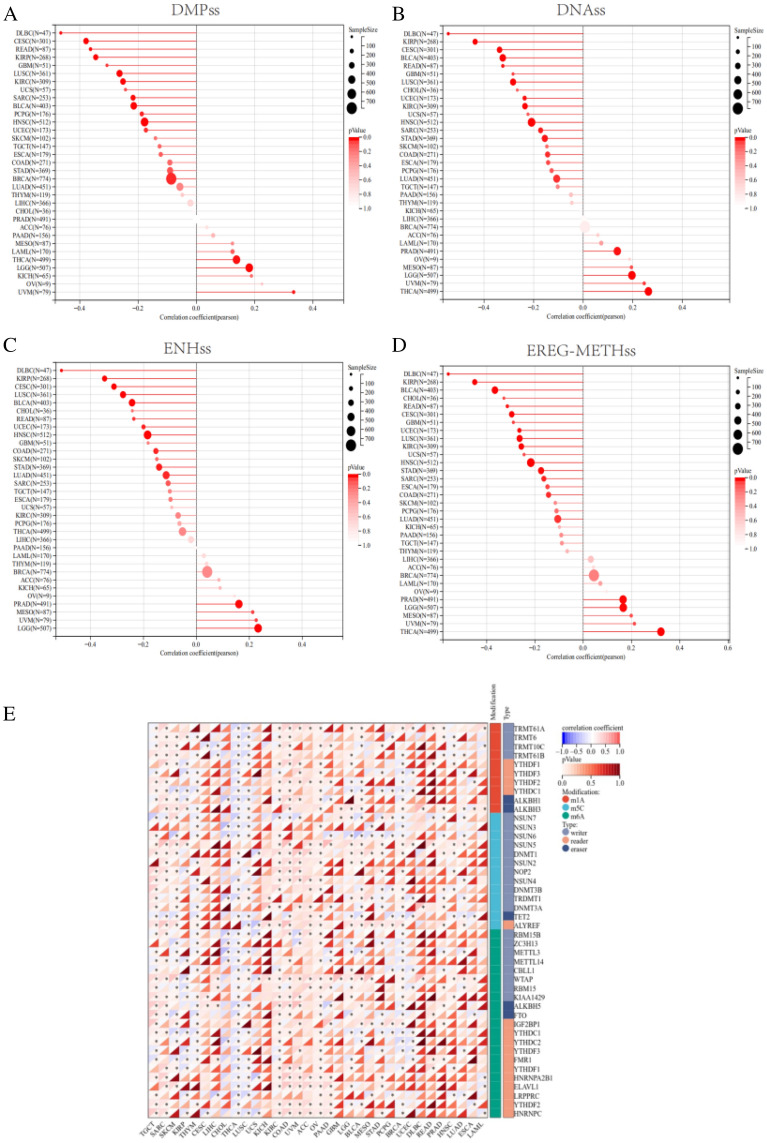
Results from the perspective of tumor stemness and epigenetic regulation. The correlation between tumor stemness and IL32 level using (**A**) DMPss, (**B**) DNAss, (**C**) ENHss, and (**D**) EREG-METHss, deeper color indicates a strong correlation. (**E**) The correlation of IL32 expression and RNA modification genes. Red and blue colors indicate positive and negative correlations, respectively; deeper color indicates a strong correlation, *P < 0.05.

Our findings revealed negative correlations between several tumors (CESC, LUAD, COAD, KIRP, STAD, UCEC, HNSC, LUSC, READ, BLCA and DLBC) stemness and IL32 mRNA expression, which was associated with poor prognosis (Fig. 8A–D). According to our findings, RNA methylation occurs most frequently in OVand COAD, followed by KIRC, and KICH, GBM and DLBC has almost no RNA methylation at the IL32 site. The results are illustrated in Fig. 8E.

### Enrichment analysis of IL32-related partners

**Figure 9:**
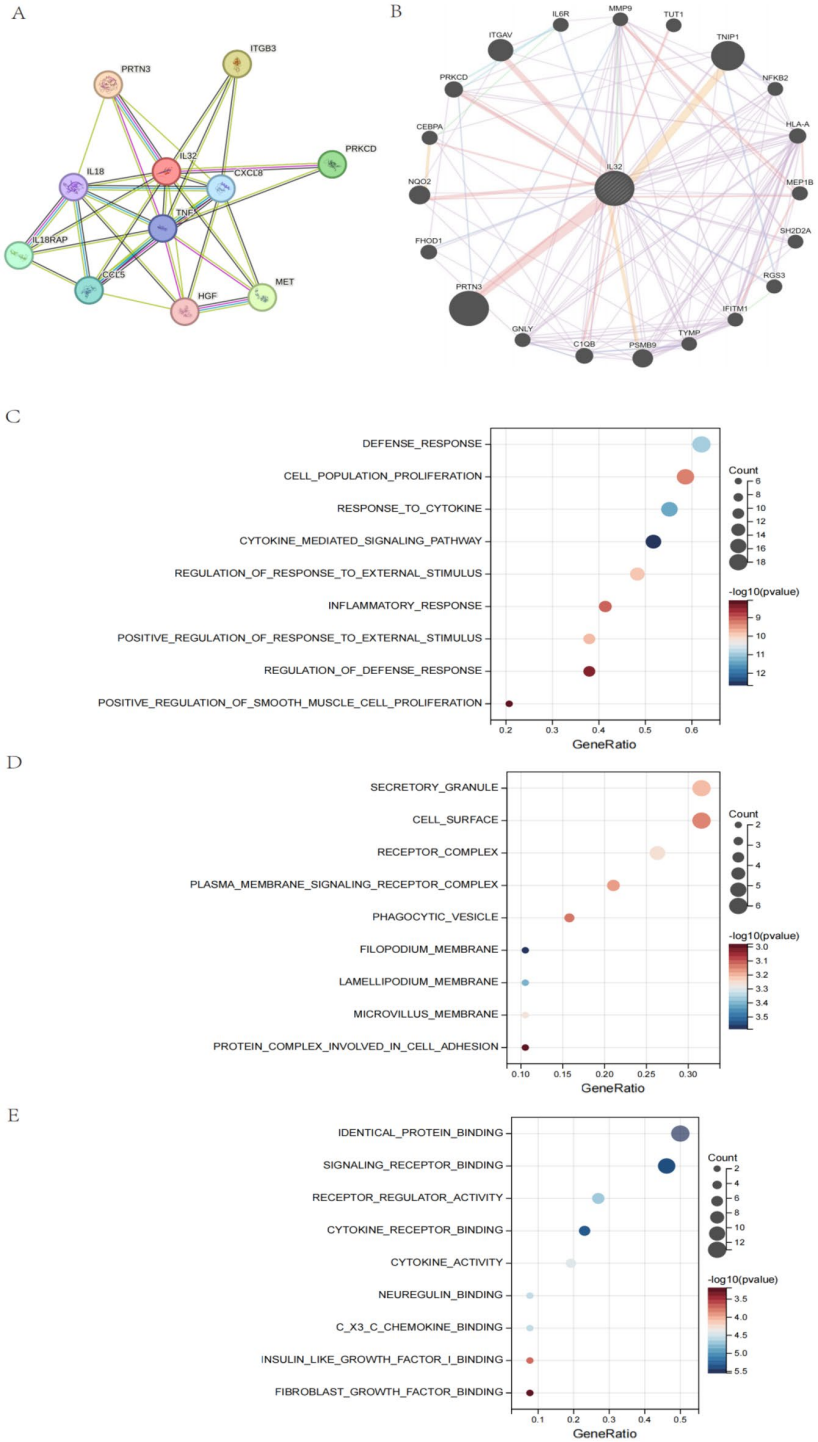
IL32-related gene enrichment analysis. (**A**) We obtained the available experimentally determined IL32-binding proteins using the STRING database. (**B**) We used the GeneMANIA website to get the 20 genes most closely related to IL32. (**C**) The biological process, (**D**) molecular function, and (**E**) cell components involved in IL32 in GO enrichment analyses.

A total of 11 proteins that interact with IL32, verified by experimental evidence, were obtained through the STRING database. The protein interaction network was shown in Fig. 9A. On the GeneMANIA website, 20 proteins that interact with IL32 were discovered and displayed in Fig. 9B.

We combined two sets of gene lists to perform GO enrichment analysis to reveal the biological process (BP), molecular function (MF), and cellular component (CC) involved in IL32. The BP involved in IL32 and its related genes covered the regulation of Defense response、Cell population proliferation and Cytokine (Fig. 9C). The main MF of IL32 included Signaling receptor binding, Receptor regulator activity and Cytokine receptor binding (Fig. 9D). The CC related to IL32 include Secretory granule and Cell surface (Fig. 9E).

## Discussion

IL-32 is an inflammatory cytokine that plays a role in the human immune system and has garnered extensive attention in cancer research in recent years. IL-32 is typically highly expressed in inflammatory diseases and various malignant tumors, inducing the release of cytokines and playing a crucial role in the formation of the inflammatory microenvironment, thereby promoting the growth, migration, and invasion of tumor cells^16–18^. However, the role of IL-32 in pan-cancer remains uncertain. This study explores the relationship between IL-32 expression and prognosis, the tumor immune microenvironment, genetic mutations, RNA methylation, immunoregulatory effects, and related signaling pathways in different tumors through pan-cancer analysis. This research aids in better understanding the role of IL-32 in the onset and development of tumors, offering clues for the development of new cancer treatment strategies.

Our research results indicate that IL32 is significantly overexpressed in 25 out of 33 cancer types, with a decrease in expression observed only in THCA and KICH. The increase in IL32 expression is associated with tumor staging, lymph node metastasis, and distant metastasis in PRAD, KIRC, PAAD, and THCA. Previous reports have suggested that high IL32 expression can promote the proliferation and metastasis of tumor cells, such as in PRAD^19^, PAAD^20^, and THCA^21^. We found that in PAAD, UVM, ACC, LGG, MESO, and GBM, high expression of IL32 is related to worse prognosis in terms of OS, DSS, PFI, and DFI. Somatic mutations can cause developmental disorders, and the gradual accumulation of mutations in the body can induce cancer^22^. Specific gene mutations are important biomarkers for patient prognosis and are beneficial for evaluating the efficacy of drug treatments for cancer patients^23,24^. IL32 mRNA expression is positively correlated with high CNA of IL32. The chromosome deletion of IL32 is most pronounced in BLCA, while chromosome amplification is most significant in BRCA. These findings imply that IL32 is an important oncogene and a potential biomarker for poor prognosis in various cancers.

Our research findings reveal that the expression of IL32 is positively correlated with the infiltration levels of DCs, CD4 T cells, CD8 T cells, and activated NK)cells. Previous studies have shown that IL-32 can activate the NF-κB signaling pathway, promote the expression of inflammation-related cytokines and chemokines (such as TNF-α, IL-6, and IL-1β), enhancing the growth and invasion capabilities of primary tumors^25,26^. However, it also attracts immune cells such as CD8 + T cells^27^, NK cells^28^, and macrophages^28^ to the tumor tissue, and can enhance the immune response of T cells and NK cells, having both anti-tumor and pro-inflammatory effects. Ohmatsu et al.^29^, found that the expression of IL-32 in sarcoidosis is positively correlated with the elevation of IDO and IL-10, possessing the ability to promote DC differentiation, foster the development of DCs or macrophages, leading to immune dysregulation and resulting in tumor immune escape.

The expression of IL32 is positively correlated with most immunosuppressants, immunostimulants, and MHC molecules. With the breakthroughs in tumor-related immunosuppressants, immunostimulants, and MHC molecules, immune checkpoint inhibitors (ICIs) have been widely applied in tumor immunotherapy and have achieved significant results^30^. IL-32 can promote the expression of programmed death ligand 1 (PD-L1) in macrophages through the PFKFB3-Januse kinase 1 (JAK1) axis, thereby promoting immune evasion^31^. The expression of PD-L1 can induce a strong immune response in tumor-infiltrating immune cells. Meanwhile, PD-1/PD-L1 ICIs have been approved for the treatment of various malignant tumors, including melanoma, lymphoma, LUAD, HNSC, KIRC, and LIHC^32,33^. Therefore, it is reasonable to speculate that the expression of IL32 may regulate the immune response of tumor immune cells and affect the prognosis of tumor patients. However, more preclinical and clinical trials are needed to explore the relationship between IL32 expression and immune checkpoints.

Research has discovered that TMB is a pan-cancer genomic biomarker, with the level of TMB being related to the generation of immunogenic peptides and the efficacy of ICIs. Furthermore, high levels of TMB contribute to enhancing the efficacy of ICIs and overall survival^34,35^. Our study shows that the expression of IL32 is associated with TMB in BRCA, ESCA, SARC, KIRP, and STAD, and with MSI in THCA, PRAD, STAD, and LUSC. The high expression of IL32 in tumors, associated with MSI and TMB, indicates that IL32 could be a valuable prognostic biomarker favorable for immunotherapy. RNA methylation plays an important role in regulating gene expression, RNA stability, RNA splicing, and the translation process^36^. RNA methylation is crucial in normal cell development and differentiation, but its abnormal regulation is associated with the development of various types of tumors^37^. In this study, there is evidence that in most common malignancies, the level of RNA methylation of IL32 is upregulated, consistent with the elevated expression of IL32. Further research is needed on the genetic alterations of IL32 as well as the relationship between RNA methylation and the expression of IL32 in cancer.

To further clarify the role and specific mechanisms of IL32 in various cancers, we utilized GO enrichment analysis to predict the speculated biological functions and related signaling pathways of IL32 in pan-cancer. Our GO enrichment study indicates that IL32 is positively correlated with cytokines and immune-related pathways. Tsai et al.^38^, reported that IL-32 promotes the invasion and metastasis of lung adenocarcinoma through the expression of matrix metalloproteinases 2 and 9 induced by NF-κB. Wang et al.^39^, demonstrated that IL-32 increases tumor growth and reduces apoptosis in breast cancer. The plasma expression levels of IL-32 in patients with malignant esophageal cancer increased, promoting the progression of esophageal cancer through the activation of NF-κB and the increased levels of cytokines TNF-α, IL-6, and IL-1β^40^. IL-32 dose-dependently accelerates the proliferation of cutaneous T-cell lymphoma^41^. IL-32 stimulates the invasion and metastasis of osteosarcoma cells through MMP-13 expression mediated by the AKT pathway. IL32 plays a crucial role in tumor development, but its potential mechanisms remain elusive, which is related to the expression subtypes of IL-32 in different tumors.IL32 has multiple isoforms, such as IL-32α, IL-32β, IL-32γ, and IL-32δ, and their specific roles and mechanisms vary in different cancers. Kang et al.^42^ reported thatThe paragraph you provided is in Chinese. IL-32α is involved in the progression of hepatocellular carcinoma, but the overexpression of IL-32α enhances natural killer cell-mediated killing by upregulating the expression of Fas and UL16 binding protein 2 (ULBP2) in human chronic myelogenous leukemia cells, exerting an anti-cancer effect^43^. IL-32γ is associated with the development of gastric cancer induced by Helicobacter pylori^44^, but in cervical cancer, IL-32γ increases apoptosis in the Hela cell line by inducing JNK signal transduction and upregulating FasL expression^45^. The roles of IL-32 isoforms in cancer suggest that they may become new therapeutic targets or biomarkers for cancer diagnosis and prognosis evaluation. Further understanding of the regulatory mechanisms of IL-32 and its isoforms in the progression of malignant tumors is of great significance for clinical judgment of tumor prognosis and treatment strategies. Therapeutic strategies targeting specific IL-32 isoforms, such as using neutralizing antibodies, small molecule inhibitors, or gene editing technologies to regulate their expression or activity, could be developed to inhibit tumor development or enhance the immune system’s ability to fight against tumors. However, realizing these potential applications requires more basic research and clinical trials to validate the roles and clinical value of IL-32 isoforms in different cancers.

In summary, this study utilized comprehensive bioinformatics analysis methods to explore the expression levels of IL32 in pan-cancer, its potential diagnostic and prognostic value, gene mutations, RNA methylation, immunoregulatory effects, and related signaling pathways. The results indicate that overexpression of IL32 has certain diagnostic value in various types of cancer. Furthermore, IL32 may be a potential prognostic and immune-related biomarker for cancer patients. This study elucidates the role of IL32 in tumor development from multiple perspectives, providing some strategies for further research into the specific mechanisms of IL32 in cancer progression and treatment.

## Data Availability

The datasets presented in this study can be found in online repositories. The names of the repository/repositories and accession number(s) can be found in the article.
